# Biomechanical analysis of patients with mild Parkinson’s disease during indoor cycling training

**DOI:** 10.1186/s12984-025-01657-1

**Published:** 2025-06-04

**Authors:** Seonghyun Kang, Jin-Woo Park, Yekwang Kim, Juhui Moon, Yeji Lee, Chan-Nyoung Lee, Jaewook Kim, Seung-Jong Kim, Byung-Jo Kim

**Affiliations:** 1 Department of Biomedical Engineering, Korea University College of Medicine, Seoul, Korea; 2 Department of Neurology, Korea University Medicine, Seoul, Korea; 3 Division of Clinical Pharmacology, Department of Medicine, Vanderbilt Medical Center, Nashville, TN USA; 4 BK21 FOUR Program in Learning Health Systems, Korea University, Seoul, Korea

**Keywords:** Parkinson’s disease, Cycling ability, Postural instability, Balance strategy, Biomechanics, Physical function assessment

## Abstract

**Background:**

Parkinson’s disease (PD) is characterized by significant postural instability and gait impairments, yet many individuals with PD can continue cycling even with severe gait dysfunction. While previous research has investigated the preservation of pedaling ability, how individuals with PD regulate and adapt their balance control strategies during cycling remains largely unexplored. This study aims to identify the biomechanical adaptations in cycling balance control employed by individuals with PD and how they differ from those of healthy individuals.

**Methods:**

A total of 39 PD patients and 42 age-matched healthy controls participated in a cycling task using a steerable indoor cycling system that enables sliding and tilting motions, requiring them to actively maintain balance while following a straight-line trajectory. Cycling dynamics were analyzed using a sensor-equipped system designed to capture medio-lateral balance adjustments, including force exertion on the handlebars and saddle, lateral deviations, and pedaling speed.

**Results:**

PD patients exhibited a higher coordination of upper and lower body in the medio-lateral direction (PD: 0.47 ± 0.18 vs. Control: 0.11 ± 0.30, *p* < 0.001), suggesting a stronger reliance on a leaning strategy for balance control. While PD patients cycled at a significantly lower freely chosen speed (6.49 ± 1.45 km/h vs. 10.28 ± 3.00 km/h, *p* < 0.001), their bike deviation was lower than that of healthy controls (PD: 17.1 ± 9.9 mm vs. Control: 22.8 ± 11.7 mm, *p* = 0.019), indicating a more constrained and controlled cycling pattern. Additionally, force distribution patterns and bike speed showed strong correlations with physical function measures, including lower limb strength and gait velocity.

**Conclusions:**

This study identifies distinct cycling balance adaptations in PD, providing insights into how individuals with PD regulate and modify their balance control strategies during cycling. The quantitative metrics derived from this study may offer a basis for future research exploring their potential as biomechanical markers for objective functional assessment and rehabilitation monitoring in PD.

## Introduction

Parkinson’s disease (PD) affects over 9.4 million individuals worldwide and is a leading cause of disability, arising from the degeneration of dopaminergic neurons in the basal ganglia, which results in reduced motor control and automaticity of movement [1–3]. Postural instability in PD leads to significant mobility challenges and an increased risk of falls, often requiring compensatory balance control strategies during gait and daily activities [4–6]. As PD advances, gait impairments become more pronounced, often leading to freezing of gait (FoG)—a disabling condition characterized by episodic movement blocks despite the intention to walk [7–9]. While static postural instability and gait dysfunction are frequently assessed in clinical settings, balance control is a broader motor function that extends beyond gait and plays a crucial role in other dynamic activities, including cycling.

Notably, despite severe postural instability and mobility deficits, many individuals with PD retain the ability to ride a bicycle [10–12]. Cycling engages distinct motor control mechanisms compared to walking, potentially benefiting from preserved rhythmic movement patterns, external sensory cues, and alternative balance-maintenance strategies [13, 14]. Given its motor and neurological benefits, cycling has been explored as a rehabilitative intervention for PD, with studies suggesting that cycling training can improve balance and functional mobility [15–17]. However, the precise biomechanical mechanisms underlying cycling balance control in PD remain poorly understood, and a lack of quantitative evaluation methods limits its clinical application [11, 18, 19].

Maintaining postural equilibrium while cycling involves a complex interplay of steering, leaning, and lateral body movements, which vary based on motor control adaptations, skill level, and population-specific characteristic [20]. For instance, novice cyclists tend to synchronize leaning with steering, while experienced cyclists decouple these actions [21]. Additionally, older cyclists rely more on lateral knee movements compared to younger individuals [22]. Investigating how individuals with PD adapt their balance control strategies during cycling could provide valuable insights into how they compensate for postural instability, beyond what can be assessed through traditional static or gait-based evaluations [23].

This study aims to characterize cycling balance strategies in individuals with PD compared to healthy individuals using a dynamic indoor cycling system. Equipped with specialized sensors and designed to allow sliding and tilting motion, this system enables a quantitative analysis of postural control strategies by capturing key biomechanical parameters. By identifying distinct balance adaptations in PD, such as altered force distribution between the upper and lower body and adjustments in pedaling speed, we seek to uncover potential compensatory mechanisms associated with impaired postural stability. Understanding these adaptive characteristics in PD during dynamic cycling may serve as a foundation for developing physical assessment tools and support evidence-based approaches for incorporating cycling into rehabilitation strategies for individuals with PD.

## Materials & methods

### Participants

PD patients with a modified Hoehn and Yahr (mH&Y) scale of 3.0 or lower, along with healthy control subjects, were recruited from the Neurology Clinic at Korea University Hospital for research purposes between September 2022 and January 2024. The diagnosis of PD was established using the United Kingdom Parkinson’s Disease Society Brain Bank diagnostic criteria. Additionally, healthy volunteers who exhibited normal muscle strength and balance during neurological examinations, with muscle strength evaluated using the Medical Research Council Scale at a grade of 5 indicating full strength, were included. Exclusion criteria for all participants included recent (within three months) neurological or musculoskeletal disorders that affect muscle strength and balance, as well as muscle weakness resulting from previous such conditions. Participants from both groups were aged between 50 and 79 years and were capable of riding a bicycle.

The PD patient group, all of whom were in the medication-ON state without FoG or a history of deep brain stimulation, underwent validated assessments, such as the Unified Parkinson’s Disease Rating Scale (UPDRS) Part III. Baseline physical function evaluations for both cohorts included the Berg Balance Scale (BBS), body composition analysis, isometric strength testing with surface electromyography (sEMG) of the knee extensors and flexors, ultrasonography (US) to assess muscle thickness and echo intensity, and gait analysis. Additionally, all participants were asked to subjectively report their prior bicycle experience, which was categorized into four levels (1: well-trained cyclist, 2: limited experience, 3: no prior experience, 4: unable to ride). All participants provided written informed consent, and the study complied with research ethics approved by the Korea University Institutional Review Board (IRB No.: 2022AN0420), in accordance with the Declaration of Helsinki guidelines.

### Experimental setup

#### Cycling assessment

**Fig. 1:**
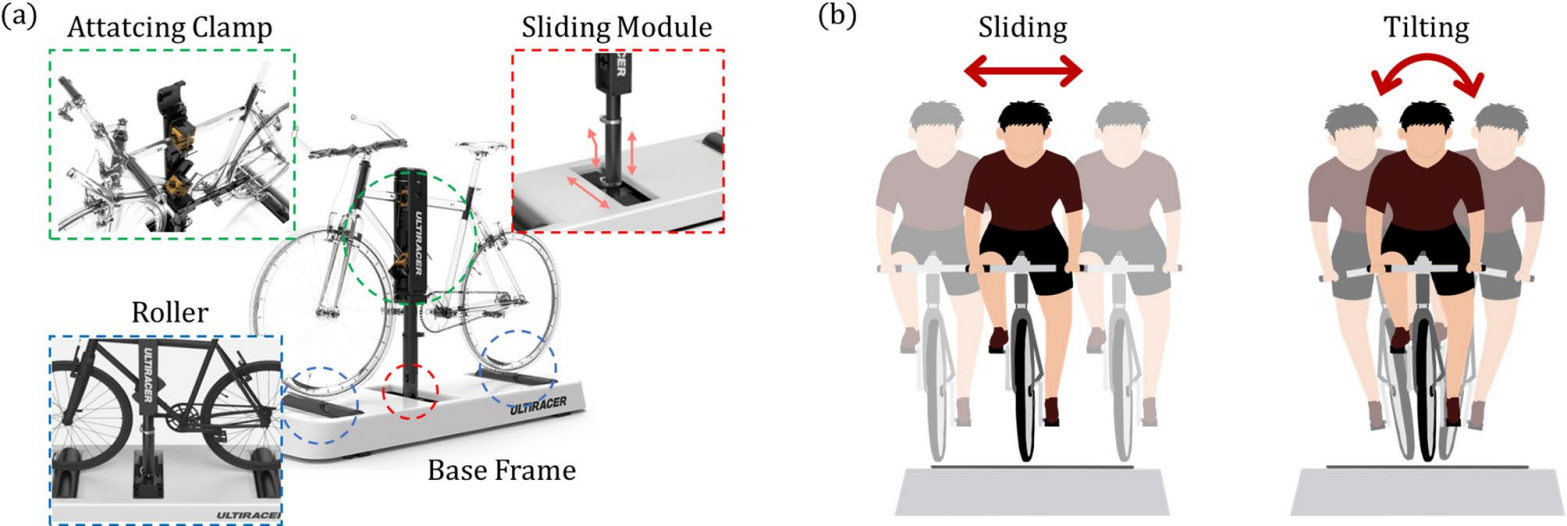
Experimental platform (**a**) Structure of the outdoor-mimicking cycling system. (**b**) Coronal plane motions, including sliding and tilting, enabled by the device

The cycling platform used in this study, the Ulti-racer P (RealDesignTech Co., Ltd, Seongnam, Korea), comprises four primary components (see Fig. 1a): a sliding module, anterior/posterior rollers, a mounting clamp, and a base frame. The sliding mounting rod, which enables medio-lateral movement and tilting capabilities (as depicted in Fig. 1b), attaches directly to the user’s bicycle using an adjustable clamp. This clamp adjusts to fit the specific dimensions of the bike, ensuring the rod aligns properly with the bike frame and that the wheels are aligned with the anterior and posterior rollers. Mechanically linked via a crank mechanism in the base frame, these rollers allow synchronized rotation and continuous contact with the bicycle’s wheels. The sliding module allows a lateral movement range of ± 10 cm from the midline and a tilting range of ± 1º. Each roller has a diameter of 110 mm, while the base frame is 520 mm wide and 1380 mm long.

**Fig. 2:**
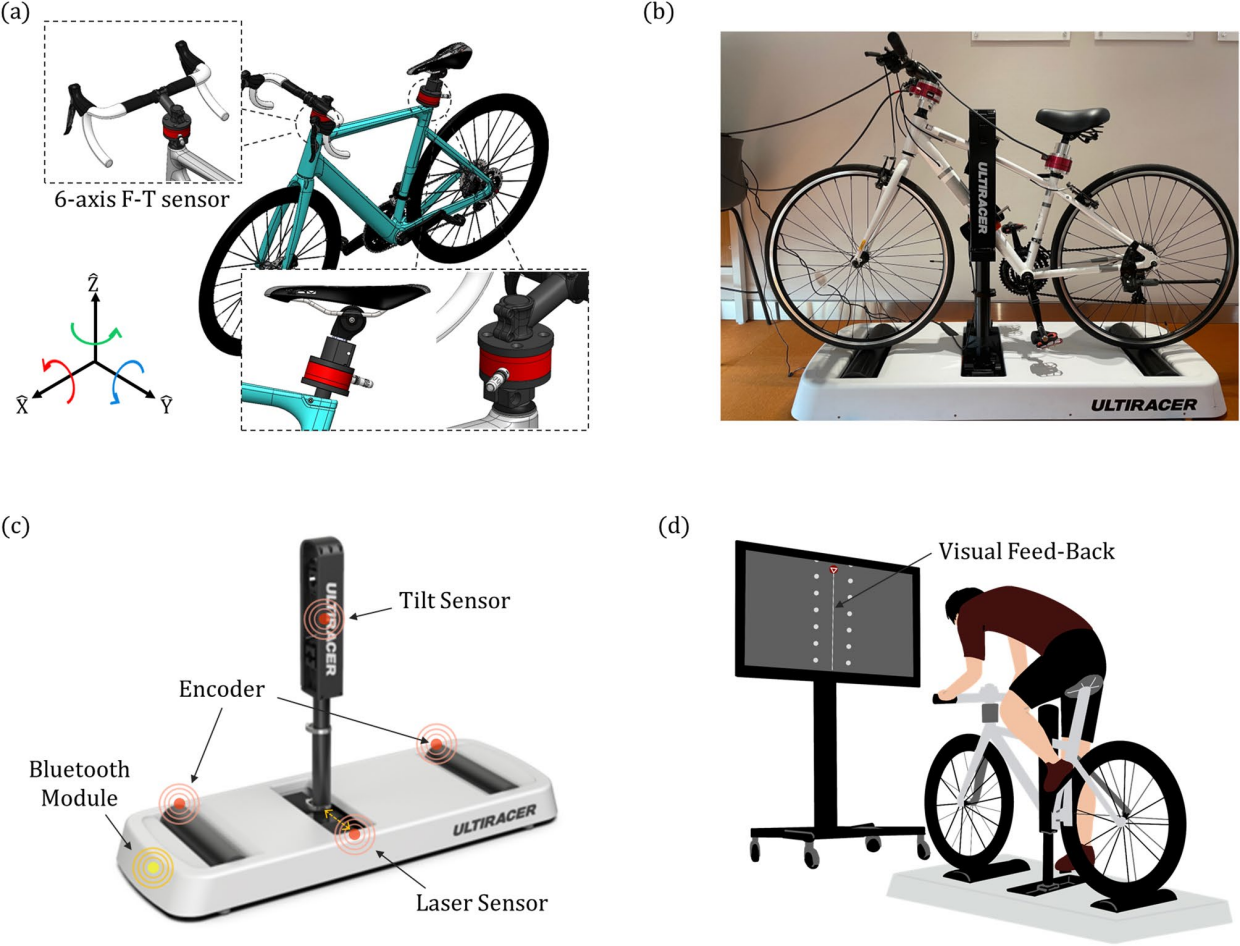
Experimental setting (**a**) Two F-T sensors were embedded in the headset space and seat post, showing their axes. (**b**) An image of the F-T sensors mounted on a bike. (**c**) Sensors equipped on the cycling platform. (**d**) Experiment setup featuring real-time visual feedback displayed on a monitor

For assessing the biomechanical aspects of cycling, sensors were integrated into both the bicycle and the platform. Two 6-axis force-torque (F-T) sensors (Dynpick, WACOH-TECH Inc., Tokyo, Japan), capable of measuring forces up to 1000 N and torques up to 30 Nm, were employed. These sensors were mounted in the headset space and seat post of the bicycle (Fig. 2a) to quantitatively measure interactions between the human and the bicycle. Additionally, an encoder on the front roller monitored speed, while an infrared distance sensor (VL53L0X, STMicroelectronics Inc., Geneva, Switzerland) positioned on the right side of the sliding module tracked the lateral movements of the rod and, consequently, the bicycle (Fig. 2c). The sampling rates were established at 50 Hz for both the distance sensor and the encoder. Meanwhile, the F-T sensor data, initially sampled at 2000 Hz, was downsampled to 200 Hz. Data were transmitted wirelessly to a computer via Bluetooth, enabling real-time display and control of an avatar within a virtual bicycle environment (Fig. 2d).

Before each experiment began, participants underwent a 10-minute acclimation period, which involved practicing straight-line riding by tracing a mid-line displayed on a monitor. They were instructed to return to the midline as quickly as possible if they deviated while riding. Adjustments to saddle height were made during this period, allowing participants to establish their preferred comfortable riding speed. After a sufficient rest interval, participants executed straight-line riding tasks, focusing on keeping the avatar centered on the mid-line for 30-second durations. This task was repeated three times, with one-minute rest intervals between sessions. Participants were instructed to grip the handlebars with both hands and maintain a steady pedaling pace as best they could. For safety, two physical therapists were stationed on each side of the participant to prevent any potential accidents.

#### Baseline physical function assessment

The sEMG data were collected from the rectus femoris (knee extensor) and biceps femoris (knee flexor) muscles using the Nicolet EDX system (Natus Neurology Inc., Middleton, WI, USA) and Synergy software (Synergy Healthcare Solutions, Maryville, TN, USA), following the methodology described in a previous study [24]. Participants were positioned prone and instructed to perform maximal knee flexion and extension against manual resistance provided by an examiner. Continuous maximal voluntary contraction (MVC) was monitored using a manual muscle tester (01165, Lafayette Instrument Company, Lafayette, LA, USA). Disposable Ag/AgCl electrodes (20 mm diameter) were placed linearly over the muscle belly with a 10 mm interelectrode distance.

For the gait assessment, participants performed three walking trials at a self-selected comfortable pace on the GAITRite^®^ system (CIR Systems Inc., Franklin, NJ, USA), a 4.27 m-long pressure-sensitive walkway equipped with sensors that detect footfalls at a sampling rate of 80 Hz. Parameters such as average step length (cm), gait velocity (cm/s), and cadence (steps/min) were recorded and averaged across the three trials.

Muscle thickness of the rectus femoris and biceps femoris was assessed on the dominant limb using B-mode ultrasound imaging (Aplio i700, Canon, Otawara, Japan) with an 18-MHz linear array transducer, following protocols from previous studies [25, 26].

### Data processing

#### Cycling data analysis

The lateral sliding distance, velocity, and 6-axis F-T data acquired from the sensors embedded in both the bicycle and the platform were initially synchronized. Idle periods between cycling sessions were excluded, followed by data concatenation from the three trials. The data were then segmented into individual cycles and normalized against the time axis, converting them into a phase-percentage format of the cycling motion.

**Fig. 3:**
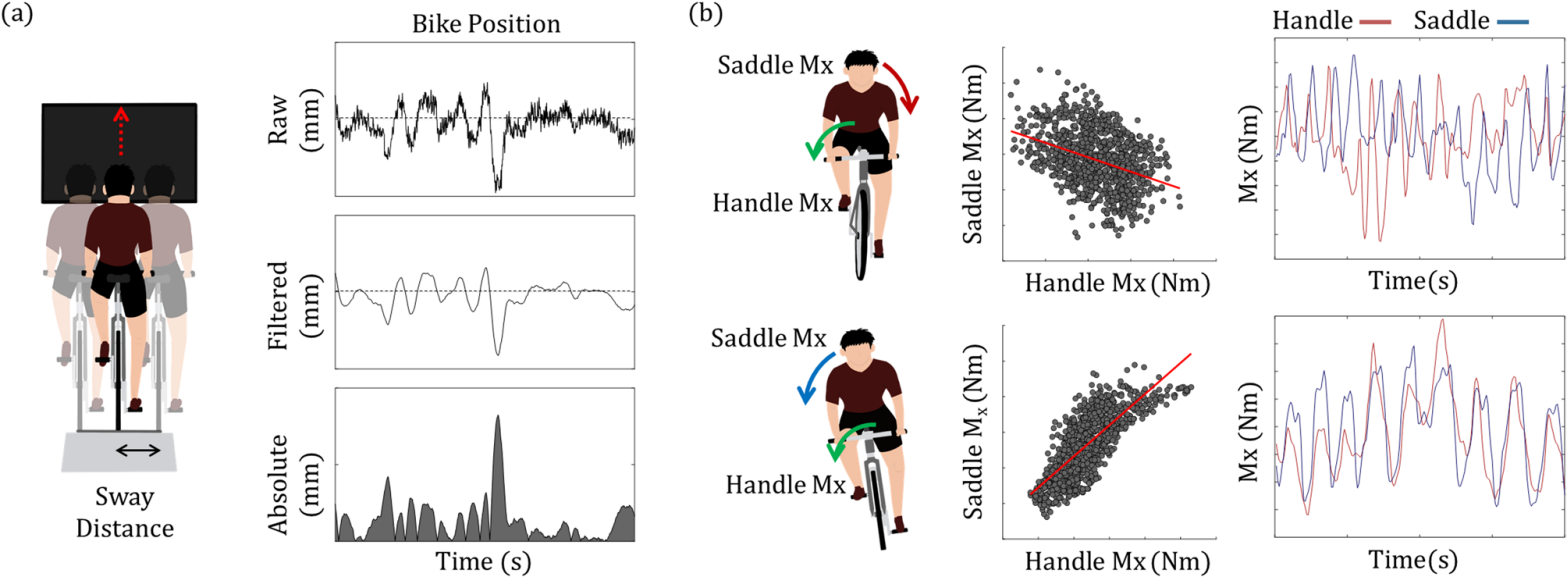
Biomechanical Features (**a**) Illustration and serial data progression for calculating the bike sway index. (**b**) Schematics for the Handle-Saddle Coordination Index (HSCI). The 1st and 2nd raw explain the negative and positive values of the moment X component (HSCI_Mx_) within the HSCI. The 2nd column displays the scatter plot of moment X (Mx) values from handle and saddle sensors (gray dots) with a polynomial fitting line (red line). The 3rd column shows the normalized time-domain Mx data from handle (red line) and saddle (blue line) sensors

We hypothesized that maintaining a straight line centered on the screen would indicate superior bicycle control ability. Therefore, the deviation distance of the bicycle from the center line was calculated (Fig. 3a). The Bicycle Sway Index (BSI) was quantified by determining two components: the root mean square ($\:{BSI}_{RMS}$) and the absolute ($\:{BSI}_{abs}$) deviation, as defined by Eqs. 1 and 2 below:

1 $$\:{BSI}_{RMS}=\sqrt{\frac{1}{{N}_{end}-{N}_{1}+1}\sum\:_{n={N}_{1}}^{{N}_{end}}{d\left(n\right)}^{2}}\:$$,

2 $$\:{BSI}_{abs}=\frac{1}{{N}_{end}-{N}_{1}+1}\sum\:_{n={N}_{1}}^{{N}_{end}}\left|d\left(n\right)\right|$$,

where $\:d\left(n\right)$ represents the distance from the middle line at frame *n*, $\:{N}_{end}$ and $\:{N}_{1}$ denote the last and initial data frames within the window for each subject, respectively.

To accurately identify and quantify the specific bicycle riding strategies employed by PD patients, we developed the Handle-Saddle Coordination Index (HSCI). This index analyzes the correlation between force and torque data captured by the 6-axis F-T sensors mounted on the bicycle’s headset and seat post (Fig. 3b). For each axis, six indices were calculated using the correlation coefficient formula specified in Eq. 3. A negative HSCI value indicates that the forces or torques exerted on the handle and saddle are in opposite directions, a pattern typically associated with the steering strategy used to maintain balance or alter riding direction. Conversely, a positive HSCI value suggests alignment in the directions of forces or moments on both the handle and saddle sensors, indicative of a leaning strategy. Equation 3 is outlined below:

3 $$\begin{array}{l}\\\:HSCI=\\\frac{\sum\:\left({f}_{handle}\left(n\right)\:-\:\stackrel{-}{{f}_{handle}\left(n\right)}\right)\:\:\left({f}_{saddle}\left(n\right)\:-\:\stackrel{-}{{f}_{saddle}\left(n\right)}\right)}{\sqrt{\sum\:{\left({f}_{handle}\left(n\right)\:-\:\stackrel{-}{{f}_{handle}\left(n\right)}\right)}^{2}\:\:\sum\:{\left({f}_{saddle}\left(n\right)\:-\:\stackrel{-}{{f}_{saddle}\left(n\right)}\right)}^{2}\:}}\:\end{array}$$,

where $\:{f}_{handle}\left(n\right)$ and $\:{f}_{saddle}\left(n\right)$ represent the force or moment data from the handle and saddle, respectively, and $\:\:\stackrel{-}{{f}_{handle}\left(n\right)}$ and $\:\:\stackrel{-}{{f}_{saddle}\left(n\right)}$ are the mean values. This HSCI formula provides a quantitative assessment of coordination between handle and saddle movements.

The Sensor Fluctuation Index (SFI) was calculated by computing the standard deviation for each axis of the F-T sensor data. We posited that this index could reflect the extent of adjustments individuals make to stabilize the bicycle trajectory. Each participant’s mean cycling velocity, chosen based on their comfort level, was also measured. Furthermore, the standard deviation of bicycle velocities was selected as a comparative parameter to identify irregularities in pedaling performance.

#### Functional assessment data analysis

Raw sEMG signals were amplified, band-pass filtered (100–500 Hz), and sampled at 48 kHz. The data were then full-wave rectified. For analysis, root mean square (RMS) values were calculated from the rectified signals over the resistance period during maximal voluntary contractions. Specifically, RMS values from the rectus femoris were used during knee extension efforts, and those from the biceps femoris were used during knee flexion.

Gait data collected from the GAITRite^®^ system were processed to compute average values of gait velocity, cadence, and step length across the three walking trials for each participant.

### Statistical analysis

Following the computation of features, a comprehensive statistical analysis was conducted to discern differences between the PD patient group and the control group. Continuous variables were presented as mean ± standard deviation in tables and depicted through box plots that displayed the median and interquartile ranges. We assessed the normality of the data using the Shapiro-Wilk test and applied unpaired t-tests to the continuous variables. Nominal variables were analyzed with Pearson’s chi-square test, and ordinal variables were evaluated using the Mann-Whitney U test.

To evaluate the discriminatory capability of biomechanical features in distinguishing PD patients from controls, univariable and multivariable logistic regression analyses were conducted using the backward Wald method. Initially, variables showing statistical significance in the univariate logistic regression—including demographic data, physical assessments, and indices derived from the cycling platform—were included. Age and gender were retained in these analyses despite not achieving statistical significance. Variance inflation factors (VIFs) were calculated to assess multicollinearity; in cases of high correlation among variables, indices derived from the cycling platform were prioritized due to their practical applicability. A refined, stepwise adjustment process was then applied to the multivariable logistic regression model (LM), ensuring that VIF values remained below 15.

To examine the relationship between physical function and features extracted from bicycle sensors, Pearson’s correlation analysis was conducted for the entire participant cohort and the PD patient group. Sensitivity and specificity of the features and the logistic regression model were assessed by analyzing the area under the receiver operating characteristic (ROC) curve. Statistical analyses were conducted using SPSS version 27 (SPSS Inc., Chicago, IL).

## Results

### Study population

A total of 82 participants were enrolled in the study, comprising 39 PD patients and 43 age-matched healthy controls. All subjects completed the experiment without any failures. Baseline demographic data, which included gender ratio, age, body mass index (BMI), and biking experience, revealed no significant differences between the two groups. However, the PD group had a significantly lower BBS score compared to the control group, with the lowest score among patients being 50. Notably, 84.6% of PD patients were in the early stages of the disease, categorized within H&Y scale stages 1 and 2. The mean UPDRS Part III score was 22.2.

Physical function assessments revealed significant differences between the two groups in isometric strength of the knee flexors, as well as in both the mean and peak sEMG RMS values of the rectus femoris muscle. Moreover, the PD group exhibited significantly slower gait velocities and shorter step lengths, as detailed in Table 1.

**Table 1:** Demographics

	Control (*n* = 43)	PD patients (*n* = 39)	p_value
Gender (male/female)	18/25	22/17	0.269
Age	65.8 ± 8.4	65.03 ± 7.3	0.672
BMI	23.3 ± 2.0	24.3 ± 3.6	0.112
Bike experience (1/2/3/4)	24/13/1/5	23/11/1/4	0.991
Berg balance scale	55.5 ± 1.0	54.4 ± 1.5	< 0.001^***^
**Parkinson related scale**			
mH&Y scale (1/2/2.5/3)		9/22/4/2	
UPDRS (III)		22.7 ± 7.9	
LEDD (mg)		326.5 ± 174.5	
**Isometric Muscle Strength (**Kg**)**			
Knee Extension	16.5 ± 5.4	14.6 ± 6.4	0.154
Knee Flexion	8.4 ± 2.9	6.7 ± 2.2	0.004^**^
**Body Composition Analysis (%)**			
Skeletal Muscle Mass (Total)	24.4 ± 4.6	25.3 ± 5.2	0.404
Trunk Muscle Mass	19.6 ± 3.3	20.5 ± 4.0	0.308
Leg Muscle Mass (Mean)	6.9 ± 1.5	7.3 ± 1.5	0.123
**sEMG** (mV)			
Extensor (Rectus Femoris)			
RMS Mean	198.5 ± 86.4	148.7 ± 110.4	0.033^*^
RMS Peak	563.5 ± 279.5	390.3 ± 280.3	0.009^**^
Flexor (Biceps Femoris)			
RMS Mean	309.1 ± 131.7	283.7 ± 153.4	0.510
RMS Peak	805.0 ± 351.5	683.0 ± 305.3	0.117
**Ultrasonography**			
Rectus Femoris			
Thickness	12.4 ± 2.5	12.0 ± 1.9	0.459
Echo Intensity	121.3 ± 47.0	112.5 ± 28.0	0.312
Biceps Femoris			
Thickness	19.8 ± 2.7	20.6 ± 3.6	0.263
Echo Intensity	249.1 ± 64.7	249.3 ± 75.8	0.987
**Gait Analysis**			
Velocity (cm/sec)	120.3 ± 13.9	106.0 ± 18.8	< 0.001^***^
Cadence (steps/min)	141.1 ± 150.9	110.5 ± 11.1	0.210
Step Length (cm)	61.3 ± 6.3	56.7 ± 9.1	0.009^**^

Unpaired t-test, Chi-square, and Mann-Whitney U tests were applied based on the type of variables (^*^p < 0.05, ^**^p < 0.01, ^***^p < 0.001). (*BMI: body mass index*,* mH&Y scale: modified Hoehn and Yahr scale*,* UPDRS: Unified Parkinson’s Disease Rating Scale*,* LEDD: Levodopa Equivalent daily dose*,* sEMG: surface electromyography*,* RMS: root mean square*)

### Group analysis of biomechanical features

**Fig. 4:**
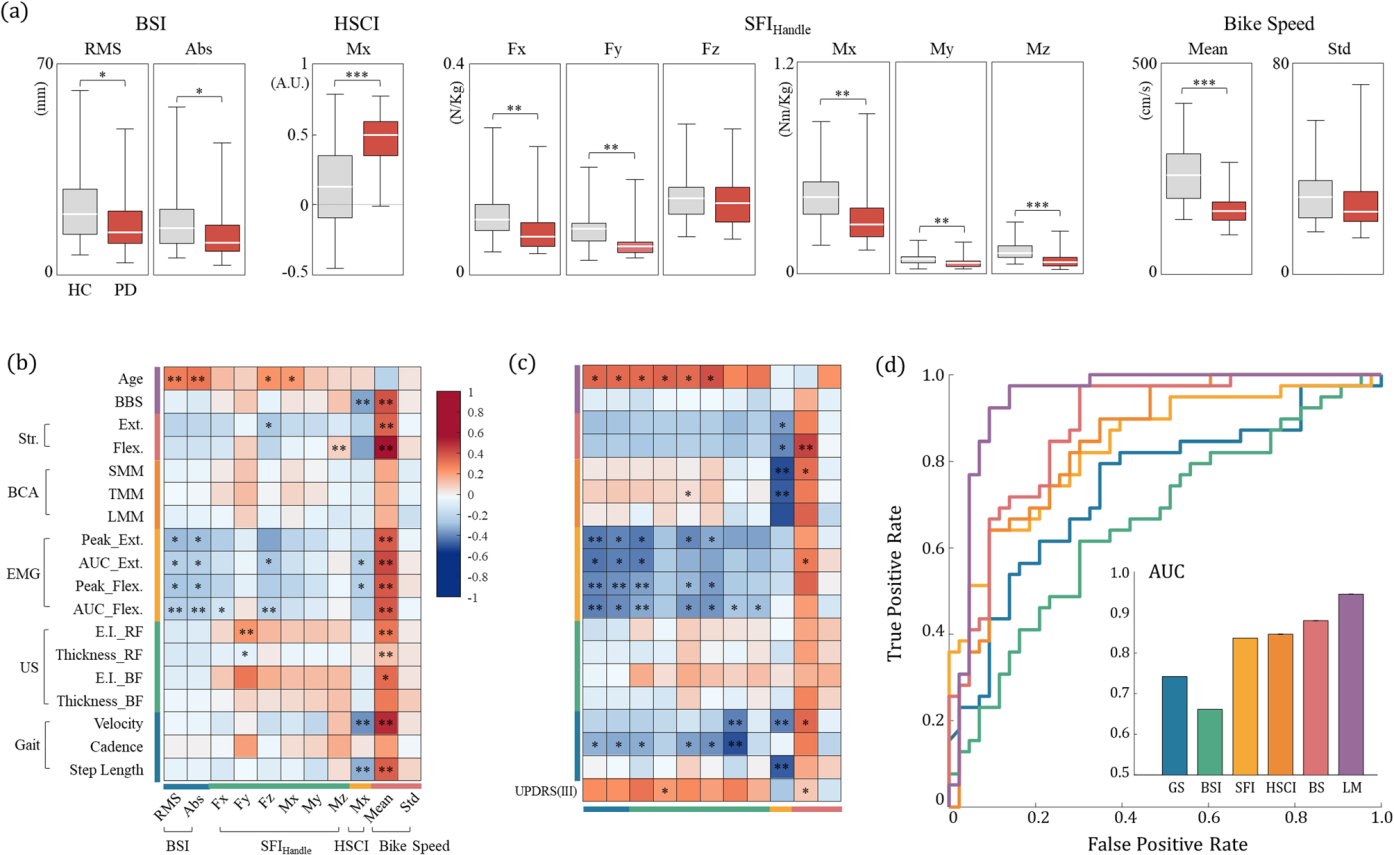
Statistical analysis of biomechanical features. (**a**) Indices generated by the cycling platform are displayed as box plots for each group, with results from the unpaired t-test (^*^p < 0.05, ^**^p < 0.01, ^***^p < 0.001). Medians are presented as white lines within the boxes, encompassing the 25th and 75th percentiles (box) and the minimum and maximum values (whiskers). Pearson’s correlation coefficients between physical assessments (rows) and biomechanical indices (columns) are depicted as heatmaps within the total population (**b**) and the PD group (**c**), respectively. Significant correlations are highlighted. (**d**) The ROC curves illustrate the sensitivity and specificity of the features and logistic regression models, while the inset bar chart displays the area under the ROC curve (AUC) values, demonstrating their diagnostic efficacy. (*BSI: bike sway index*,* RMS: root means square*,* ABS: absolute*,* HSCI: handle-saddle coordination index*,* SFI*_*handle*_: *Sensor fluctuation index of handle*,* Fx/Fy/Fz: force of x-y-z axis*,* Mx/My/Mz: moment of x-y-z axis*,* HC: healthy control*,* BBS: Berg Balance Scale*,* Str.: strength*,* BCA: Body composition analysis*,* US: ultrasonography*,* Ext.: extensor*,* Flex: flexor*,* RF: rectus femoris*,* BF: biceps femoris*)

The comparative analysis of BSI, HSCI, SFI, and cycling speed between PD patients and healthy controls is delineated through box plots in Fig. 4a and detailed statistical results in Table 2. Notably, BSI values were significantly lower in PD patients, indicating diminished deviations in their bicycle trajectory. A notable difference was observed in the x-axis moment component of HSCI (HSCI_Mx_), suggesting it as a distinct feature of cycling mechanics in PD patients. Most PD patients had positive HSCI_Mx_ values, unlike healthy controls who showed greater variability and lower mean values. This indicates a concerted application of torque by PD patients in the same direction to maintain stability. PD patients also demonstrated significantly reduced average cycling speeds. Additional detailed parameters not depicted in Fig. 4a are provided in Table 2, highlighting distinctive biomechanical traits separating PD patients from healthy controls.

**Table 2:** Group analysis for index from cycling platform

	Control (*n* = 43)	PD Patients (*n* = 39)	*P* Value
**Bike Sway Index** (mm)			
RMS	22.8 ± 11.7	17.1 ± 9.9	0.019^*^
Absolute Value	18.3 ± 10.5	13.7 ± 8.7	0.034^*^
**H-S Coordination Index**			
Force X	-0.24 ± 0.30	-0.25 ± 0.22	0.943
Y	0.06 ± 0.20	0.13 ± 0.27	0.187
Z	0.27 ± 0.25	0.31 ± 0.21	0.381
Moment X	0.11 ± 0.30	0.47 ± 0.18	< 0.001^***^
Y	0.10 ± 0.22	0.04 ± 0.24	0.250
Z	-0.21 ± 0.18	-0.17 ± 0.22	0.248
**Sensor Fluctuation Index**			
Handle			
Force X	0.114 ± 0.046	0.086 ± 0.049	0.009^**^
Y	0.088 ± 0.037	0.061 ± 0.033	0.001^**^
Z	0.150 ± 0.044	0.141 ± 0.051	0.352
Moment X	0.072 ± 0.025	0.054 ± 0.028	0.002^**^
Y	0.014 ± 0.005	0.011 ± 0.005	0.005^**^
Z	0.021 ± 0.008	0.012 ± 0.007	< 0.001^***^
Saddle			
Force X	0.264 ± 0.080	0.256 ± 0.066	0.620
Y	0.237 ± 0.065	0.230 ± 0.052	0.594
Z	0.401 ± 0.144	0.444 ± 0.200	0.266
Moment X	0.037 ± 0.009	0.038 ± 0.008	0.563
Y	0.049 ± 0.020	0.049 ± 0.017	0.944
Z	0.024 ± 0.007	0.020 ± 0.008	0.055
**Bike Speed**			
Average Speed (cm/s)	285.6 ± 83.3	180.3 ± 40.2	< 0.001^***^
Standard Deviation (Fluctuation)	30.1 ± 10.1	27.5 ± 11.6	0.276

Statistical analysis was conducted using an unpaired t-test (^*^p < 0.05, ^**^p < 0.01, ^***^p < 0.001)

### Correlation analysis of Biomechanical features and physical assessments

Pearson’s correlation analysis was conducted to explore the relationship between physical assessment tests and sensor-derived features, including the PD group, as depicted in Fig. 4b and c, respectively. The analysis revealed that BSI_RMS_ exhibited weak positive correlations with participant age and negative correlations with sEMG values for both knee extensor and flexor muscles. This pattern persisted even when the analysis was confined to PD patients. Additionally, HSCI_Mx_ showed significant negative correlations with the BBS, gait velocity, and step length. Specifically in the PD group, body composition and isometric strength inversely correlated with HSCI_Mx_. Conversely, speed demonstrated strong positive correlations with BBS, gait velocity, cadence, and strength-related parameters, especially involving knee flexors. The UPDRS Part III revealed weak positive correlations with the z-axis force component (Fz) of the SFI value from the handle sensor (SFI_handle_) and bicycle speed, suggesting that disease severity may influence balancing strategies in PD patients. Detailed correlation coefficients and p-values are available in Supplementary Tables 1 and 3.

In the PD group, the unadjusted Pearson correlation between HSCI and gait metrics was *r* = − 0.413, *p* = 0.009 (Fig. 4c). Given that gait speed is a well-established indicator of physical function in PD, we conducted a more detailed analysis to examine its association with HSCI. To account for potential confounding factors, we performed a multiple regression analysis adjusting for disease severity (UPDRS score), muscle strength (isometric knee extensor strength), and baseline characteristics (age, BBS, and BMI). After adjustment, the correlation was attenuated but remained statistically significant (*r* = − 0.333, *p* = 0.038), indicating that HSCI remains a notable association with gait speed, even after controlling for these factors (Fig. S1).

**Fig. 5:**
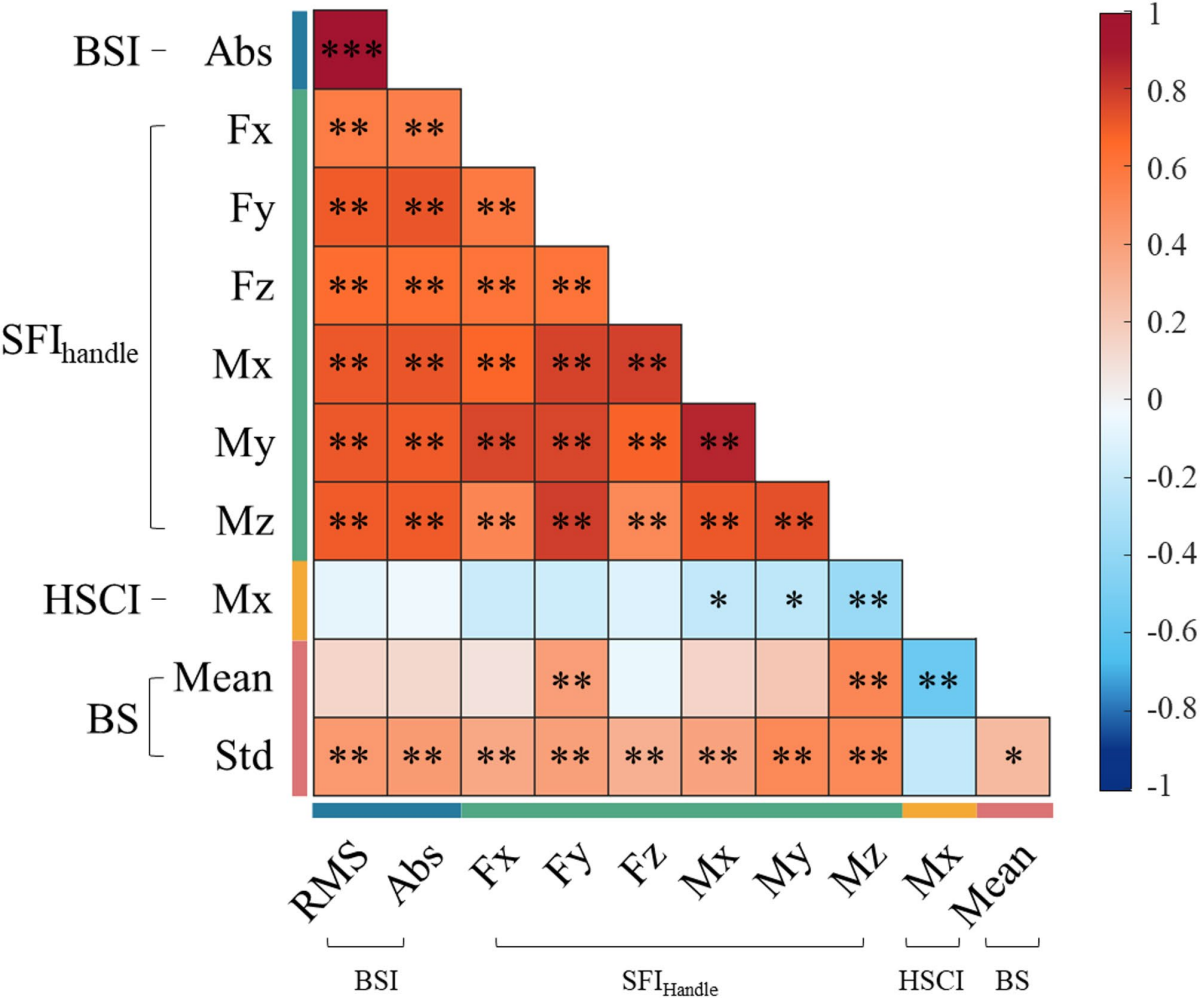
Correlation analysis between bike indices. The heatmap illustrates Pearson’s correlation coefficients among indices derived from the cycling platform for all participants. Significant correlations are denoted with asterisks (^*^p < 0.05, ^**^p < 0.01, ^***^p < 0.001)

Despite evident statistical differences between the two groups, understanding the underlying biomechanics requires analysis of the relationships among these indices. Figure 5 and Supplementary Table 2 present the results of the Pearson’s correlation analysis for indices across all participants. A prominent high correlation was observed between BSI_RMS_ and all SFI_handle_ components, along with a weak positive correlation with the standard deviation of bicycle speed. The SFI_handle_ values also exhibited a strong positive inter-correlation. HSCI_Mx_ displayed low negative correlations with the moment values of SFI_handle_ and moderate negative correlations with mean bicycle speed, presenting a correlation coefficient of -0.545 (*p* < 0.001). The adjusted R-squared value was 0.289 for the relationship between mean bicycle speed and HSCI_Mx_, and 0.495 for the relationship between BSI_RMS_ and the Mz component of SFI_handle_.

### Logistic regression model and ROC curve analysis

To assess the capability of quantitative biomechanical cycling characteristics to distinguish PD patients from healthy controls, we conducted ROC curve and area under cover (AUC) analyses (Fig. 4d). We evaluated feature specificity and sensitivity, including gait velocity, a well-known discriminative parameter for PD, for comparative analysis. Our results showed that bicycle speed had the highest AUC value of 0.880, with a 95% confidence interval (CI) ranging from 0.806 to 0.953. Additionally, HSCI_Mx_ (AUC: 0.846, 95% CI: 0.762–0.930) and SFI_handle_Mz_ (AUC: 0.837, 95% CI: 0.749–0.924) also demonstrated robust performance in differentiating PD patients.

To improve discriminative capability, we designed a multivariate binary logistic regression model using features found significant in univariate analysis. These variables were further refined through the backward Wald method (Table 3) to develop the final LM, which included HSCI_Mx_, average bicycle speed, SFI_handle_Fx_, and gender. Concerns about interpretation arose from the limited value ranges of HSCI_Mx_ (-0.452 to 0.783) and SFI_handle_Fx_ (0.040 to 0.280), which led to inflated odds ratios. To achieve consistency, we standardized these values by dividing HSCI_Mx_ by 10 and SFI_handle_Fx_ by 100, resulting in an adjusted odds ratio of 1.550 and 0.183, respectively. A 0.1 unit increase corresponds to a 1.550-fold increase in the odds, and a 0.01 unit increase corresponds to a 0.183-fold increase.

The logistic model achieved an accuracy of 0.914, precision of 0.907, recall of 0.929, and specificity of 0.897. Multicollinearity tests indicated a VIF of 10.565, well below the threshold of 15, suggesting no concerns about multicollinearity. The ROC curve for the multivariate model showed an AUC of 0.945 (95% CI: 0.889-1.000), reflecting superior performance relative to the analysis of individual features (Fig. 4d).

**Table 3:** Logistic regression analysis

Univariate Analysis						
	β	S.E.	Odds Ratio (e^β^)	95% CI for Exp(B)		*p*_value
				Lower	Upper	
**Gender**	0.586	0.447	1.797	0.748	4.317	0.190
**Flex. Strength**	-0.271	0.1	0.763	0.627	0.928	0.007^**^
**Ext. RMS Mean**	-0.005	0.003	0.995	0.989	1.000	0.043^*^
**Gait Velocity**	-0.055	0.017	0.946	0.916	0.977	0.001^**^
**BSI**_**RMS**_	-0.052	0.024	0.949	0.906	0.994	0.026^*^
**SFI**_**handle**_ **_Fx**	-14.267	5.854	< 0.001	< 0.001	0.061	< 0.001^***^
**HSCI_Mx**	6.121	1.419	455.158	28.201	7346.228	< 0.001^***^
**Mean Bicycle Speed**	-0.031	0.007	0.969	0.956	0.983	< 0.001^***^
**Multivariate Analysis**						
**HSCI _Mx**	4.380	1.987	79.817	1.625	3.921 × 10^3^	0.028^*^
**Mean Bicycle Speed**	-0.033	0.009	0.967	0.949	0.985	< 0.001^***^
**SFI**_**handle**_ **_Fx**	-16.959	7.460	< 0.001	< 0.001	0.097	0.023^*^
**Gender Identity**	2.120	0.869	8.327	1.515	45.767	0.015^*^
**Constant Value**	6.355	2.451	575.642			0.010^**^

The backward Wald method was utilized for logistic regression analysis (^*^p < 0.05, ^**^p < 0.01, ^***^p < 0.001). (*BSI: bike sway index*,* SFI: sensor fluctuation index*,* HSCI: handle-saddle coordination index*)

## Discussion

Individuals with PD retain their cycling ability despite severe gait impairment, a phenomenon that has often been explored by analyzing the differences between gait and pedaling to determine its underlying cause. Rather than taking this approach, our study focuses on understanding how individuals with PD maintain balance while cycling, emphasizing balance control strategies in dynamic cycling rather than simply the retention of ability. To investigate this, we systematically analyzed the biomechanical properties and balance adaptations of PD patients during dynamic cycling, comparing these characteristics to those of age-matched healthy controls. Our findings indicate that PD patients exhibit a significantly stronger coupling between upper and lower body force distribution and a slower self-selected pedaling speed, resulting in a more constrained cycling pattern. Additionally, these biomechanical features correlated with traditional physical function measures and demonstrated strong discriminatory performance in distinguishing PD from controls when applying adjusted models, suggesting their potential for future use in quantitative assessments tailored for PD patients.

The BSI serves as an indicator of lateral deviations during cycling while attempting to maintain a straight path. This index shows a negative correlation with EMG parameters and a positive correlation with age, for both the PD and healthy control groups, suggesting its potential role in assessing age-related physical functions. Interestingly, contrary to our expectations of increased postural instability in PD patients, our findings reveal that individuals with PD exhibit lower BSI values compared to their healthy elderly counterparts. This may be explained by specific cycling strategies adopted by the PD group, including significantly reduced cycling speed and an increased HSCI, which likely serve as compensatory mechanisms for stability. Individuals characterized by postural instability and motor impairments, such as those with PD, sarcopenia, and other elderly populations, adopt alternative gait patterns involving slower speeds, shorter step lengths, and wider step widths [27–29]. While these adaptations may reduce gait efficiency, they enhance stability during walking. Therefore, we believe the lower cycling speed and BSI observed in the PD group may be interpreted as a compensation pattern consistent with gait strategies aimed at enhancing stability.

While BSI reflects lateral deviations and potential compensatory strategies for maintaining a straight path, HSCI provides further insights into how PD patients regulate balance through force application at the handlebars and saddle. Previous studies have elucidated control strategies for maintaining bicycle equilibrium, including steering, leaning, and lateral knee movements, which are influenced by a blend of physical and cognitive functions [22, 30]. In our experimental setup, the x-axis moment recorded by the saddle F-T sensor indicates the lateral leaning control of the upper body. To capture steering actions, we utilized the x-axis moment of the handlebar F-T sensor, since the z-axis component, typically used to measure handlebar rotation, tends to yield less conspicuous values. By calculating correlation coefficients between the x-axis moments of the handlebar and saddle F-T sensor values, we sought to elucidate the relationship between leaning and steering strategies. The results show that HSCI_Mx_ values are significantly elevated in PD patients, suggesting that they maintain balance by synchronously applying force to both the handlebars and saddle in the same direction. In contrast, healthy elderly individuals achieve balance either by applying antagonistic forces to the handlebars and saddle in a compensatory manner or by managing these forces independently without coupling. Although investigations into PD patients’ cycling strategies are limited, the existing literature suggests that novice cyclists often exhibit a pronounced integration of leaning and steering actions, whereas expert cyclists demonstrate more independent control methods [21, 31]. This observation suggests that the compromised postural stability or diminished physical performance observed in PD patients may manifest in distinct cycling techniques.

Moreover, although the HSCI_Mx_ values showed no direct correlation with UPDRS part III scores, a significant correlation with gait parameters was observed. This lack of alignment with UPDRS may stem from its structure: despite being a robust tool for evaluating the severity and physical function of PD patients, it includes only 2 of the 18 items in the Motor-III assessment questionnaires related to postural instability and gait dysfunction [23]. Hence, the strong correlation with gait parameters, even after adjusting for confound factors, implies that HSCI_Mx_ might effectively serve as a quantitative indicator for assessing a patient’s postural instability and physical function.

Results from the ROC curve and AUC analysis indicate significant differences in postural instability among PD patients. The univariate parameters’ AUC values show that mean cycling speed, HSCI_Mx_, and SFI_handle_ are crucial in distinguishing PD patients from controls, displaying AUC values even higher than that of gait velocity. Using features from the cycling platform and baseline demographic data in a multivariate binary LM, the accuracy reached 91.4% with an AUC value of 0.945. Although this study does not primarily aim to enhance the binary classification performance of PD from controls, the simple logistic model provides comparable results to previous studies. Additionally, logistic regression analysis reveals that HSCI_Mx_ and mean cycling speed are significant variables with high odds ratios even after covariate adjustment, indicating the substantial impact of these biomechanical features. Although moderate correlations exist between HSCI_Mx_ and cycling speed, suggesting that the specific control strategy may arise from cycling speed rather than being an exclusive characteristic of PD, the absence of multicollinearity in the logistic regression and the relatively low R-square value indicate the independence of the two variables.

This study offers clinical insights into balance impairments in PD, showing that patients adopt constrained cycling strategies to compensate for postural instability. These adaptations underscore the potential of cycling-based interventions for dynamic balance training and assessment. The strong discriminatory performance of key features and their correlation with traditional functional measures suggest that cycling evaluations could serve as valuable complementary tools for motor assessments, aiding clinical decision-making. For example, they may help determine when a PD patient has sufficient functional stability to safely engage in outdoor cycling as part of rehabilitation. Additionally, the steerable indoor cycling system used in this study provides a controlled and sufficiently challenging environment, making it a promising platform for both training and assessment. For PD patients who are not yet capable of cycling outdoors, this system offers a safe and dynamic rehabilitation tool, enabling them to practice balance control while simultaneously undergoing objective assessment. While this study primarily aimed to explore biomechanical adaptations, it lays the groundwork for future research to validate its clinical applications in rehabilitation and assessment.

This study has limitations related to the selected PD cohort, experimental conditions, and statistical analysis. As a preliminary study and the first to apply the steerable indoor cycling system in PD patients, we prioritized an early-stage PD population, limiting the inclusion of late-stage patients with FoG or those in a medication-OFF state. Future studies should examine the applicability of these findings to patients with more severe symptoms and explore correlations with disease progression. Additionally, cycling sessions were restricted to a single straight-path ride at a self-selected speed. Future research should incorporate more complex riding scenarios, such as turns, obstacle avoidance, balance recovery after perturbations, and varied pedaling speeds, to better simulate real-world cycling challenges and further distinguish PD patients from healthy individuals. Expanding the experimental design could also clarify whether the observed adaptations stem from cognitive deficits or motor impairments. Regarding statistical analysis, correlation analyses involved multiple comparisons without correction, as this study aimed to observe general trends rather than validate quantitative indices. Thus, results should be interpreted with caution. Future studies should adopt study designs and statistical methods tailored to evaluating these biomechanical markers’ clinical relevance and validity.

## Conclusion

This study identifies distinct balance control strategies in PD during cycling, offering insights into how individuals with PD adapt to impaired postural stability. Our findings demonstrate that PD patients adopt a lower self-selected cycling speed and a more tightly coupled force distribution between the upper and lower body, distinguishing their balance regulation from that of healthy individuals. While the primary aim of this study was to explore biomechanical adaptations, these findings also lay the groundwork for future research into their potential as objective markers for quantitative functional assessment in PD. Additionally, although further validation is needed, the steerable indoor cycling system presents a promising platform for both assessment and rehabilitation, as its ability to integrate real-time evaluation with balance training may contribute to improved functional outcomes in PD.

## Data Availability

The raw data supporting the findings of this study are available upon request to the corresponding author.

## Abbreviations

PD: Parkinson’s disease
FoG: Freezing of gait
mH&Y: Modified Hoehn and Yahr
UPDRS: Unified Parkinson’s Disease Rating Scale
BBS: Berg Balance Scale
sEMG: Surface electromyography
US: Ultrasonography
F-T: Force-torque
HSCI: Handle-Saddle Coordination Index
HSCI_Mx_: x-axis moment component of HSCI
BSI: Bike Sway Index
BSI_RMS_: Root mean square value of BSI
BSI_abs_: Absolute value of BSI
SFI: Sensor Fluctuation Index
SFI_handle_: SFI value from the handle sensor
Fx/Fy/Fz: x/y/z-axis force
Mx/My/Mz: x/y/z-axis moment
RMS: Root mean square
ROC: Receiver operating characteristic
VIF: Variance inflation factor
LM: Logistic regression model
BMI: Body mass index
AUC: Area under cover

## References

[1] Kim J, Porciuncula F, Yang HD, Wendel N, Baker T, Chin A, et al. Soft robotic apparel to avert freezing of gait in Parkinson’s disease. Nat Med. 2024;30(1):177–85. 38182783 10.1038/s41591-023-02731-8

[2] Park JW, Kwon DY, Choi JH, Park MH, Yoon HK. Olfactory dysfunctions in drug-naïve Parkinson’s disease with mild cognitive impairment. Parkinsonism Relat Disord. 2018;46:69–73. 29233469 10.1016/j.parkreldis.2017.11.334

[3] Yuan RY, Chen SC, Peng CW, Lin YN, Chang YT, Lai CH. Effects of interactive video-game–based exercise on balance in older adults with mild-to-moderate Parkinson’s disease. J Neuroeng Rehabil. 2020;17(1):91. 32660512 10.1186/s12984-020-00725-y PMC7359629

[4] Mirelman A, Herman T, Nicolai S, Zijlstra A, Zijlstra W, Becker C, et al. Audio-biofeedback training for posture and balance in patients with Parkinson’s disease. J Neuroeng Rehabil. 2011;8(1):35. 21693054 10.1186/1743-0003-8-35 PMC3142211

[5] Kwon KY, You J, Kim RO, Lee EJ, Lee J, Kim I, et al. Association between baseline gait parameters and future fall risk in patients with de Novo Parkinson’s disease: forward versus backward gait. J Clin Neurol. 2024;20(2):201–7. 38171499 10.3988/jcn.2022.0299 PMC10921052

[6] Kwon KY, Park S, Lee HM, Park YM, Kim J, Kim J, et al. Backward gait is associated with motor symptoms and fear of falling in patients with de Novo Parkinson’s disease. J Clin Neurol. 2019;15(4):473–9. 31591835 10.3988/jcn.2019.15.4.473 PMC6785475

[7] Lo AC, Chang VC, Gianfrancesco MA, Friedman JH, Patterson TS, Benedicto DF. Reduction of freezing of gait in Parkinson’s disease by repetitive robot-assisted treadmill training: a pilot study. J Neuroeng Rehabil. 2010;7(1):51. 20946640 10.1186/1743-0003-7-51 PMC2972300

[8] Park H, Shin S, Youm C, Cheon SM, Lee M, Noh B. Classification of Parkinson’s disease with freezing of gait based on 360° turning analysis using 36 kinematic features. J Neuroeng Rehabil. 2021;18(1):177. 34930373 10.1186/s12984-021-00975-4 PMC8686361

[9] Kim SJ, Paeng SH, Kang SY. Stimulation in supplementary motor area versus motor cortex for freezing of gait in Parkinson’s disease. J Clin Neurol. 2018;14(3):320–6. 29856153 10.3988/jcn.2018.14.3.320 PMC6032003

[10] Aerts MB, Abdo WF, Bloem BR. The bicycle sign for atypical parkinsonism. Lancet. 2011;377(9760):125–6. 21215882 10.1016/S0140-6736(11)60018-4

[11] Snijders AH, Toni I, Ruzicka E, Bloem BR. Bicycling breaks the ice for freezers of gait. Mov Disord. 2011;26(3):367–71. 21462254 10.1002/mds.23530

[12] Snijders AH, Bloem BR. Cycling for freezing of gait. N Engl J Med. 2010;362(13). 10.1056/NEJMicm0810287 20357278

[13] Chang HC, Lu CS, Chiou WD, Chen CC, Weng YH, Chang YJ. An 8-week low-intensity progressive cycling training improves motor functions in patients with early-stage Parkinson’s disease. J Clin Neurol. 2018;14(2):225–33. 29629527 10.3988/jcn.2018.14.2.225 PMC5897207

[14] McGough EL, Robinson CA, Nelson MD, Houle R, Fraser G, Handley L et al. A tandem cycling program: feasibility and physical performance outcomes in people with Parkinson disease. J Neurol Phys Ther. 2016;40(4). 10.1097/NPT.0000000000000146 27576091

[15] Tiihonen M, Westner BU, Butz M, Dalal SS. Parkinson’s disease patients benefit from bicycling - a systematic review and meta-analysis. NPJ Parkinsons Dis. 2021;7(1):86. 34561455 10.1038/s41531-021-00222-6 PMC8463550

[16] Arcolin I, Pisano F, Delconte C, Godi M, Schieppati M, Mezzani A, et al. Intensive cycle ergometer training improves gait speed and endurance in patients with Parkinson’s disease: a comparison with treadmill training. Gait Posture. 2016;34(1):125–38. 10.3233/RNN-150506 26684265

[17] Ferraz DD, Trippo KV, Duarte GP, Neto MG, Santos KOB, Oliveira Filho JJ, et al. The effects of functional training, bicycle exercise, and exergaming on walking capacity of elderly patients with Parkinson disease: a pilot randomized controlled single-blinded trial. Am J Phys Med Rehabil. 2018;99(5):826–33. 10.1016/j.apmr.2017.12.014 29337023

[18] Licen T, Rakusa M, Bohnen NI, Manganotti P, Marusic U. Brain dynamics underlying preserved cycling ability in patients with Parkinson’s disease and freezing of gait. Front Physiol. 2022;13:847703. 10.3389/fpsyg.2022.847703 PMC9244145 35783714

[19] Yi J, Soudbakhsh D, Zhang Y, Zhang Y, editors. Why some Parkinson’s disease patients cannot stand or walk but can ride a bicycle: a control system-based analysis. In: Dynamic Systems and Control Conference; 2012; American Society of Mechanical Engineers.

[20] Vlakveld WP, Twisk D, Christoph M, Boele M, Sikkema R, Remy R, et al. Speed choice and mental workload of elderly cyclists on e-bikes in simple and complex traffic situations: a field experiment. Accid Anal Prev. 2015;74:97–106. 25463949 10.1016/j.aap.2014.10.018

[21] Cain SM, Ashton-Miller JA, Perkins NC. On the skill of balancing while riding a bicycle. PLoS ONE. 2016;11(2). 10.1371/journal.pone.0149340 PMC4766243 26910774

[22] Bulsink VE, Kiewiet H, van de Belt D, Bonnema GM, Koopman BJ. Cycling strategies of young and older cyclists. Hum Mov Sci. 2016;46:184–95. 26796419 10.1016/j.humov.2016.01.005

[23] Penko AL, Hirsch JR, Voelcker-Rehage C, Martin PE, Blackburn G, Alberts JL. Asymmetrical pedaling patterns in Parkinson’s disease patients. Clin Biomech (Bristol Avon). 2014;29(10):1089–94. 10.1016/j.clinbiomech.2014.10.006 PMC4362538 25467810

[24] Park JW, Baek SH, Sung JH, Kim BJ. Predictors of step length from surface electromyography and body impedance analysis parameters. Sens (Basel). 2022;22(15):5686. 10.3390/s22155686 PMC9371228 35957243

[25] Sung JH, Baek SH, Park JW, Rho JH, Kim BJ. Surface electromyography-driven parameters for representing muscle mass and strength. Sens (Basel). 2023;23(12):5490. 10.3390/s23125490 PMC10302141 37420659

[26] Baek SH, Sung JH, Park JW, Son MH, Lee JH, Kim BJ. Usefulness of muscle ultrasound in appendicular skeletal muscle mass Estimation for sarcopenia assessment. PLoS ONE. 2023;18(1). 10.1371/journal.pone.0280202 PMC9844922 36649288

[27] Osoba MY, Rao AK, Agrawal SK, Lalwani AK. Balance and gait in the elderly: a contemporary review. Lancet Neurol. 2019;4(1):143–53. 10.1002/lio2.252 PMC6383322 30828632

[28] Perez-Sousa MA, Venegas-Sanabria LC, Chavarro-Carvajal DA, Cano-Gutierrez CA, Izquierdo M, Correa-Bautista JE, et al. Gait speed as a mediator of the effect of sarcopenia on dependency in activities of daily living. J Cachexia Sarcopenia Muscle. 2019;10(5):1009–15. 31066999 10.1002/jcsm.12444 PMC6818451

[29] Skiadopoulos A, Moore EE, Sayles HR, Schmid KK, Stergiou N. Step width variability as a discriminator of age-related gait changes. J Neuroeng Rehabil. 2020;17:1–13. 32138747 10.1186/s12984-020-00671-9 PMC7059259

[30] Dubbeldam R, Baten C, Buurke J, Rietman JS. SOFIE, a bicycle that supports older cyclists? Accid Anal Prev. 2017;105:117–23. 27745781 10.1016/j.aap.2016.09.006

[31] Kovacsova N, de Winter JC, Schwab AL, Christoph M, Twisk D, Hagenzieker MJ, et al. Riding performance on a conventional bicycle and a Pedelec in low-speed exercises: objective and subjective evaluation of middle-aged and older persons. Transp Res F Traffic Psychol Behav. 2016;42:28–43.

